# Cognitive training at home for clinically defined insomnia: effects on sleep and psychological functioning

**DOI:** 10.3389/fdgth.2026.1725773

**Published:** 2026-02-02

**Authors:** Jose L. Tapia, Jon Andoni Duñabeitia, F. Javier Puertas

**Affiliations:** 1 Facultat de Psicologia i Logopèdia, Universitat de València, Valencia, Spain; 2 Centro de Investigación Nebrija en Cognición (CINC), Universidad Nebrija, Madrid, Spain; 3 Unidad Sueño, Hospital Universitario de la Ribera-FISABIO, Valencia, Spain; 4 Facultad de Medicina, Universidad Católica de Valencia, Valencia, Spain

**Keywords:** cognitive training, digital therapeutics, emotional well-being, executive function, insomnia, sleep quality

## Abstract

**Introduction:**

Insomnia is widespread globally and is often maintained by dysfunctional cognitive-emotional processes. This open-label feasibility study examined whether participation in a home-based computerized cognitive training (CCT) program implemented via a commercial platform was associated with changes in sleep and related outcomes in adults from the general population reporting persistent sleep difficulties.

**Methods:**

Thirty-two adults completed a four-week CCT intervention delivered via the CogniFit platform. The program consisted of 20 self-guided training sessions (∼45 min each), combining cognitive tasks targeting attention, memory, and executive functioning. Standardized self-report questionnaires and a computerized cognitive battery were administered immediately before and after the intervention to assess sleep quality, mood, cognition, and quality of life. Changes over time were examined using repeated-measures analyses, with Gender included as an exploratory between-subjects factor.

**Results:**

Relative to baseline, post-intervention assessments showed statistically significant reductions in insomnia severity and sleep complaints, along with decreases in depressive symptoms and worry. Changes were also observed in executive functioning and global cognitive performance. No significant change was found in self-reported quality of life. Exploratory analyses indicated gender-related differences in depressive symptoms, worry, and executive functioning.

**Conclusions:**

In this community sample, participation in a cognitively oriented computerized training program was associated with changes in sleep-related, emotional, and cognitive measures over time. Given the single-group feasibility design and modest sample size, these findings should be interpreted as preliminary and do not allow conclusions regarding clinical efficacy. Larger randomized controlled trials are needed to determine whether such cognitively focused interventions provide benefits beyond non-specific effects and to clarify their role as adjuncts within multimodal approaches to insomnia.

## 1 Introduction

Insomnia disorder (ID) is a pressing public health concern worldwide, with prevalence rates estimated between 3.9% and 22.1% (1–3). Defined by chronic difficulties initiating or maintaining sleep despite adequate opportunities, ID significantly increases the likelihood of both psychiatric (e.g., depression, anxiety, bipolar disorder) (4) and non-psychiatric conditions (e.g., Alzheimer's disease, type 2 diabetes) (5, 6). Recent perspectives propose that ID is best viewed as a transdiagnostic phenomenon, suggesting overlapping etiological links with numerous neuropsychiatric syndromes (7). One prominent explanation is the hyperarousal hypothesis, which asserts that individuals with ID experience heightened autonomic, endocrine, and neurophysiological activity that disrupts sleep onset and continuity (8).

Cognitive Behavioral Therapy for Insomnia (CBT-i) is currently considered the first-line treatment for insomnia disorder and is supported by robust empirical evidence (9, 10). Meta-analytic findings indicate that CBT-i typically produces moderate reductions in insomnia severity and related sleep complaints, although treatment response shows substantial interindividual variability and may attenuate over long-term follow-up (11, 12). To address barriers related to cost, time, availability of trained professionals, and limited implementation within healthcare systems, digital adaptations of CBT-i (dCBT-i) have been developed to improve scalability and access. Evidence from large randomized trials and meta-analyses suggests that dCBT-i can yield clinically meaningful improvements in insomnia symptoms comparable to face-to-face formats in the short term, although adherence and sustained engagement remain important challenges in real-world settings (13–15). Notably, both traditional and digital CBT-i appear to be less effective for certain biological and psychophysiological presentations of insomnia, such as objectively short sleep duration and persistent cognitive-emotional hyperarousal (16–18). These limitations have motivated growing interest in complementary or adjunctive interventions that may address neurocognitive mechanisms implicated in insomnia maintenance, including deficits in executive functioning and impaired inhibitory control (19–21).

Cognitive training, which involves structured tasks designed to strengthen cognitive functions, offers a theoretically motivated approach to addressing neurocognitive processes implicated in sleep regulation. A growing body of experimental research suggests that intensive cognitive activity can influence sleep architecture through the close relationship between learning, memory consolidation, and sleep (22, 23). These effects are thought to be mediated, in part, by the modulation of neural circuits such as the default mode network (DMN), which is associated with memory consolidation and the regulation of brain activity during rest and sleep. In the context of insomnia, a condition characterized by dysfunctions in the DMN and patterns of cortical hyperactivation, cognitive training may act as a modulator to restore functional balance in these circuits (24, 25). This not only enhances memory consolidation processes but also facilitates emotional regulation during sleep, contributing to greater stability in sleep quality. Empirical support for this hypothesis comes primarily from experimental research. For example, a single one-hour, one-hour session of visuomotor adaptation training administered in the morning has been found to increase slow-wave activity (SWA) during subsequent nocturnal sleep compared to a no-training control condition (23), with comparable effects reported when similar training is administered late in the evening (26). Likewise, administering a multicomponent cognitive training before a daytime nap has been associated with improvements in sleep continuity, stability, and organization among young adult samples (27). While these laboratory-based interventions provide valuable implications about the relationship between daytime cognitive activity and sleep architecture they lack ecological validity as they are conducted under highly controlled experimental setting that do not capture the complexity of real-world sleep behaviors and environmental influences. As a result, they do not fully explore how such training might influence daily functioning or long-term sleep quality in naturalistic settings.

Against this background, computerized cognitive training (CCT) represents a logical extension of this experimental work by translating cognitive training paradigms into ecologically valid digital formats that can be implemented in everyday environments. CCT programs are typically developed as commercial tools aimed at cognitive enhancement rather than as clinical interventions, yet a substantial body of research has demonstrated that such programs can yield small-to-moderate but reliable improvements in general cognitive functioning both in healthy and clinical populations (28–30). However, only a limited number of studies have specifically examined whether engagement with CCT may also be associated with changes in sleep-related outcomes. A pioneering randomized controlled trial demonstrated that an 8-week CCT program significantly improved sleep quality in older adults with insomnia. In that study, 51 participants were assigned either to a cognitive training group (*n* = 34) or to an active control group (*n* = 17), with the training group showing earlier sleep onset, reduced wake time after sleep onset, fewer nocturnal awakenings, and improved sleep efficiency, as assessed using actigraphy and sleep diaries (31). Consistent with these findings, a large randomized group-based study in a community-dwelling older adult population (*N* = 420) reported significant post-intervention improvements in subjective sleep quality following an 8-week multicomponent CCT program, compared with a control condition (32). More recently, a clinical feasibility study extended the scope of CCT research beyond sleep-related outcomes by also examining emotional well-being and everyday functioning in a clinical population. In that study, participants who completed an intensive 15-day CCT regimen showed improvements in sleep quality, reductions in anxious-depressive symptoms, and enhanced daily functioning and activities of daily living, as assessed through self-administered questionnaires (33). However, as this sample consisted of patients recruited from a hospital-based sleep unit who presented severe sleep disturbances and notable impairments in daily functioning, the generalizability of these findings to broader populations remains unclear. To advance the field, it is therefore essential to establish whether CCT can yield similar benefits in individuals who experience persistent sleep disturbances but without formal diagnoses. This population represents a large segment of the community whose sleep complaints are meaningful yet often remain unrecognized or untreated, making it a critical target for scalable, accessible interventions.

In light of the limited and heterogeneous evidence on the application of computerized cognitive training to sleep-related outcomes, the present pilot study aimed to examine whether participation in a self-guided, home-based CCT program is associated with changes in sleep and related domains in adults from the general population reporting persistent sleep difficulties. We examined changes in insomnia severity and subjective sleep quality as primary outcomes, alongside secondary outcomes related to emotional functioning (depressive symptoms and worry), cognitive performance (executive functioning and global cognition), and quality of life. Given the single-group feasibility design and the exploratory nature of the study, no causal claims were formulated; instead, the findings are intended to inform the feasibility, scope, and outcome sensitivity of cognitively oriented digital interventions for sleep difficulties, and to guide the design of future randomized controlled trials.

## 2 Materials and methods

This study employed a convenience-based, self-selected sampling design consistent with its open-label, single-group feasibility and proof-of-concept nature. Recruitment was conducted online between March and June 2023 through local press, institutional social media, and pharmacy-based advertisements in the Comunidad Valenciana (Spain). No formal *a priori* sample size calculation was performed, as the primary aim of the study was to assess feasibility, adherence, and outcome sensitivity rather than to test intervention efficacy.

The registration process included a screening questionnaire with the following inclusion criteria: (a) aged between 18 and 65 years; (b) experiencing difficulties in falling asleep, maintaining sleep, or waking up early without being able to fall back asleep; (c) these difficulties occur even when conditions for sleep are favorable; (d) these difficulties occur at least three nights per week; (e) these difficulties have been present for at least three months; (f) these difficulties cause distress or interfere with daily functioning. Exclusion criteria included: (a) working night shifts; (b) consuming more than 150 mg of caffeine per day or more than 250 mL of alcohol per day. These criteria were used as general indicators of the presence of a sleep disturbance consistent with insomnia, ensuring the sample reflected individuals experiencing meaningful sleep difficulties. Additionally, the Epworth Sleepiness Scale, the Restless Legs Syndrome Questionnaire, and the STOP-BANG Questionnaire were administered to rule out sleep difficulties influenced by these conditions; participants scoring higher than 11, 2, or 3 on these respective scales were excluded. No further exclusion criteria were applied regarding psychiatric comorbidities or the use of sleep-related medication. Concomitant treatments were neither initiated, modified, nor standardized by the study team. This decision was made to retain a sample that reflects the naturalistic clinical profile of individuals who typically report persistent sleep difficulties, in whom mood or anxiety symptoms and ongoing medication use are highly prevalent and were therefore treated as part of the real-world context of the study rather than controlled factors (1). Sociodemographic data were also collected online as part of the registration.

Participants who met the criteria were redirected to the CogniFit mobile application (CogniFit Inc., San Francisco, US) for evaluation and training. Upon registration, participants signed an e-consent and were granted access to the study, while those who did not meet the criteria were notified accordingly. After initiation, a neuropsychologist from the research team contacted participants to verify the accuracy of the information provided and ensure suitability for the participation; however, this brief verification call did not involve a structured clinical interview or any formal diagnostic assessment. No clinical diagnosis of insomnia disorder was conducted, and eligibility relied exclusively on the self-report screening procedure described above. Thus, the sample represents individuals from the general population experiencing chronic sleep complaints, rather than a clinically diagnosed insomnia disorder cohort.

The CogniFit platform was fully preconfigured to deliver the study procedures in an automated sequence. After registration, participants first completed the baseline questionnaires, which became available upon accessing the application. Once all baseline assessments were submitted, the platform automatically enabled access to the cognitive training program. Training sessions were released according to the programmed schedule (see Section 2.2), with built-in flexibility allowing up to two days between sessions while still maintaining the expected weekly rhythm. Participants received automated notifications when a new session was available. After completing the full sequence of training sessions, the platform unlocked the post-intervention questionnaires. This automated workflow ensured standardized administration without researcher involvement. Only participants who adhered to the full protocol were included in the final analyses.

### 2.1 Participants

**Figure 1:**
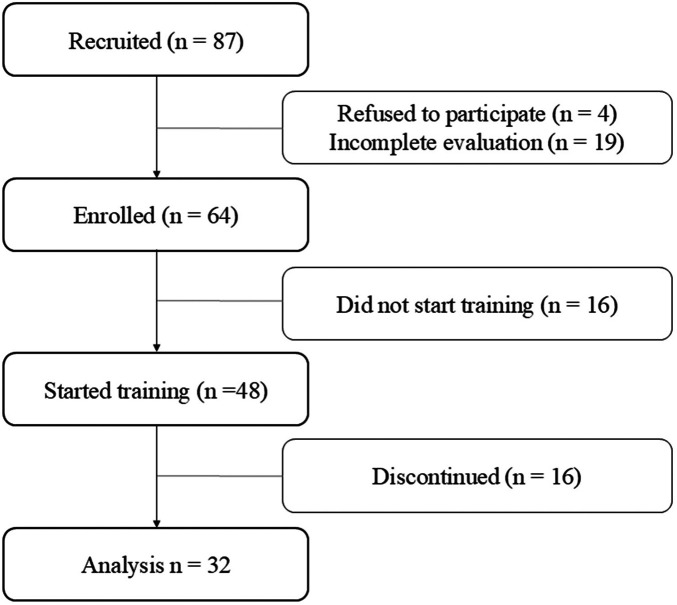
CONSORT diagram flow. Shows participant enrollment, exclusions, and completion rates throughout the study.

A total of 87 individuals enrolled in the study after meeting the inclusion criteria. Of these, 4 participants withdrew from the study, and 19 did not complete the initial evaluation, resulting in 64 eligible participants. Subsequently, 16 of these participants did not initiate the training, leaving 48 individuals who began the intervention. 16 participants did not fully adhere to the training regimen and were therefore classified as non-completers, resulting in a final sample of 32 participants who successfully completed the entire protocol (see Figure 1).

The final sample included in the analysis consisted of 32 participants, of which 21 were women (*M*_age_ = 47.7; *SD* = 7.02) and 11 were men (*M*_age_ = 49.0; *SD* = 14.0). In terms of marital status, 19 participants were married, 8 were single, 4 were divorced, and 1 was in another marital situation. Regarding educational level, 4 participants had completed primary education, 11 had completed secondary education, and 17 had higher education. Concerning the area of residence, 27 participants lived in urban areas, while 5 resided in rural areas.

Regarding the use of sleep medication, 12 participants reported taking some form of medication. Of these, 5 combined multiple medications: 3 were taking Melatonin and Lorazepam, 1 was taking Lorazepam and Doxylamine, and 1 was taking Lorazepam and Zolpidem. The remaining 7 participants took a single medication: 3 took Melatonin, 2 took Lorazepam, 1 took Lormetazepam, and 1 took Doxylamine.

Participants were compensated with a neuropsychological report and an annual license for personalized cognitive training.

### 2.2 Intervention

The intervention program comprised 20 cognitive training sessions, each lasting approximately 45 min. Participants were instructed to complete five sessions per week, with a mandatory 12-hour interval between sessions to prevent overtraining. Throughout the intervention, participants had access to email and telephone support. The research team monitored progress, establishing weekly contact with participants and additional contact if technical issues or lack of adherence were detected.

Cognitive training was delivered via the CogniFit platform, a commercially available computerized cognitive training system that has been examined in multiple empirical studies showing small-to-moderate improvements in cognitive performance, particularly in near-transfer outcomes, across healthy and clinical populations (34). Importantly, this evidence is not specific to insomnia, and CogniFit has not been validated as a stand-alone clinical treatment for sleep disorders; in the present study, the platform was used as a delivery system for standardized cognitive training tasks rather than as an insomnia-targeted digital therapeutic.

A total of 24 game-like activities were used; these are the same activities employed by Tapia et al. (33). The program targets core cognitive domains including sustained and selective attention, working memory, inhibitory control, cognitive flexibility, and processing speed. These functions are particularly relevant in the context of insomnia, as deficits in attentional control, inhibitory processes, and executive functioning have been linked to cognitive-emotional hyperarousal, rumination, and difficulties disengaging from wake-related cognitive activity (35, 36). A detailed description of all training tasks and the cognitive domains targeted by each of them is presented in Supplementary Material S1. Each activity offers instructions and includes 10 difficulty levels with 9 sub-levels. The Individualized Training System™ (ITS) automatically adjusts the difficulty based on individual performance and sequences the activities according to each user's needs, ensuring no activity is repeated consecutively. During the first session, users completed a brief practice trial; in subsequent sessions, instructions appeared as a reminder, but practice trials could be skipped. A pause button enabled users to revisit instructions, watch an explanatory video, or undertake a practice session if needed.

### 2.3 Exploratory moderators

The assessment was conducted at two time points: the day before starting the training and the day after completion, following the same structure and procedure described in Tapia et al. (33). At each time point, participants completed a standardized cognitive evaluation together with seven self-report questionnaires, administered in a fixed order across participants. Beyond primary indices of sleep quality, the assessment included measures of mood, anxiety, worry, executive functioning, global cognitive performance, and quality of life, thereby capturing a set of domains commonly affected in individuals with persistent sleep difficulties.

#### 2.3.1 Main exploratory moderators

##### 2.3.1.1 Insomnia Severity Index (ISI)

A 7-item scale evaluating sleep quality. Each item is scored from 0 to 4, with total scores ranging from 0 to 28. Higher scores reflect greater severity of insomnia (37). The total direct score was used in this study. A global score ≥ 20 is commonly used as a clinical cut-off indicating at least moderate depressive symptomatology, and a reduction of approximately 5 to 6 points is typically considered the (minimal clinically important difference) MCID for this instrument (38).

##### 2.3.1.2 Pittsburgh Sleep Quality Index (PSQI)

A 19-item scale assessing sleep habits. The scale comprises 7 components, each scored from 0 to 3. The global score, the sum of the component scores, ranges from 0 to 21, with higher scores indicating greater sleep dysfunction (39). The total direct score was used in this study. A global score ≥ 5 is the established cut-off for clinically significant sleep disturbance, and a reduction of approximately 3 points is commonly considered the MCID.

#### 2.3.2 Secondary exploratory moderators

##### 2.3.2.1 Cognitive assessment battery (CAB)®

This battery includes 17 neuropsychological tasks with a total duration of approximately 35 min. It measures 22 cognitive skills grouped into 5 cognitive domains, culminating in a general cognitive performance score. Z-scores are provided for each level, with higher scores indicating superior cognitive ability (CogniFit Inc., 2024). In this study, only the global score was considered. The instrument does not provide specific clinical cut-offs or MCID values; however, as is standard in neuropsychological assessment, z-scores ≤ −1.5 are typically interpreted as indicating clinically significant cognitive impairment (40).

##### 2.3.2.2 Beck Depression Inventory (BDI)

A 21-item scale assessing depressive symptoms. Each item is scored from 0 to 3, yielding a total score range of 0 to 63. Higher scores denote greater severity of depressive symptoms (41). The overall direct score was used in this study. Scores are commonly interpreted using standard severity ranges (0–13 minimal, 14–19 mild, 20–28 moderate, 29–63 severe depressive symptoms), and a reduction of approximately 17.5% is typically considered the MCID for this instrument (42).

##### 2.3.2.3 State-Trait Anxiety Inventory (STAI)

This inventory comprises 40 items divided into two subscales: 20 items for trait anxiety and 20 items for state anxiety (43). For this study, only the state anxiety subscale was considered. Each item is scored from 0 to 3, resulting in a total score range of 0 to 60. Higher scores indicate more pronounced anxiety symptoms. The total direct score for the state anxiety subscale was used in this study. Scores equal to or above 40 on the standard metric are commonly used as a clinical cut-off indicating clinically significant anxiety.

##### 2.3.2.4 Penn State Worry Questionnaire (PSWQ)

A 16-item scale measuring levels of worry. Each item is scored from 1 to 5, resulting in a total score range of 16 to 80. Higher scores signify higher levels of worry (44). The total direct score was used in this study. The instrument provides established clinical cut-offs scores ≥ 45 indicating clinically significant worry and scores ≥ 60 indicating severe pathological worry.

##### 2.3.2.5 Behavior Rating Inventory of Executive Function (BRIEF)

A 75-item scale divided into 9 categories, evaluating executive function impairment. Each item is scored from 0 to 2, with a total score range of 0 to 150. Higher scores represent greater interference in daily tasks (45). The total direct score was used in this study. The instrument provides normative cut-offs (T ≥ 65 indicating clinically significant impairment), no MCID has been established for this instrument.

##### 2.3.2.6 World Health Organization Quality-Of-Life Scale (WHOQOL)

A 26-item scale assessing quality of life across 4 domains and a general index. Each item is scored from 1 to 5, yielding a total score range of 26 to 130. Higher scores indicate better quality of life (46). The total direct score was used in this study. A change of approximately 2 points in WHOQOL-BREF domain scores is generally considered the MCID (47).

### 2.4 Statistical analysis

Data analysis was conducted using jamovi (48). For each outcome measure, a separate repeated-measures ANOVA was conducted to examine change over time from pre- to post-intervention. Each model included a single within-subjects factor, *Evaluation Time* (pre- and post-intervention), and *Gender* as a between-subjects factor. We report (a) the main effect of *Evaluation Time*, (b) the main effect of *Gender*, and (c) the *Time*  ×  *Gender* interaction to examine whether pre- to post-intervention changes differed between men and women. Given the unequal sample sizes and limited power to detect small gender effects, these analyses were considered exploratory. Effect sizes are reported as partial eta squared (*η*p^2^). *Post hoc* comparisons were conducted using the Bonferroni correction to identify significant differences between specific pairs of means. A *p*-value of less than.05 was considered statistically significant for all analyses. Complementary Bayesian hypothesis tests (49, 50) were conducted to quantify the strength of evidence in favor of the alternative hypothesis relative to the null hypothesis (BF₁₀) for pre- vs. post-intervention comparisons, thereby complementing frequentist analyses by estimating the degree of evidence for the presence or absence of change.

Sensitivity analyses were conducted using G*Power 3.1 software (51). Assuming an alpha level of.05, statistical power of.80, and a total sample size of 32 participants, the sensitivity analysis indicated that the design was capable of detecting effect sizes as small as *f* = 0.21 for within–between interaction effects.

All analyses were based on available data generated by the platform. Post-intervention assessments were automatically unlocked only after completion of the full training protocol; therefore, participants who did not complete the intervention did not provide post-intervention data. As a result, no additional data exclusion or imputation procedures were applied, and all pre-post comparisons reflect available-case data inherent to the study design.

## 3 Results

The results have been organized into four main categories: (1) primary measures of sleep quality, including the Insomnia Severity Index (ISI) and the Pittsburgh Sleep Quality Index (PSQI); (2) mental health measures related to sleep, such as the Beck Depression Inventory (BDI), the State-Trait Anxiety Inventory (STAI), and the Penn State Worry Questionnaire (PSWQ); (3) executive and cognitive function measures, assessed using the Behavior Rating Inventory of Executive Function (BRIEF) and the Cognitive Assessment Battery (CAB); and (4) quality of life measures, using the World Health Organization Quality-of-Life Scale (WHOQOL). Table 1 presents the descriptive statistics for all instruments before and after the intervention.

**Table 1:** Descriptive statistics for each measure (pre and post intervention).

Measure	Pre *M* (SD) [95% CI]	Post *M* (SD) [95% CI]
ISI**	16.66 (4.02) [15.21, 18.11]	14.31 (4.83) [12.57, 16.05]
PSQI*	11.75 (3.31) [10.56, 12.94]	10.88 (3.50) [9.61, 12.14]
BDI**	14.47 (9.42) [11.07, 17.86]	9.56 (6.79) [7.12, 12.01]
STAI	34.03 (12.48) [29.53, 38.53]	31.22 (9.86) [27.66, 34.77]
PSWQ*	57.84 (15.92) [52.10, 63.59]	54.00 (14.54) [48.76, 59.24]
BRIEF*	56.88 (29.16) [46.36, 67.39]	52.38 (25.67) [43.12, 61.63]
CAB**	−0.09 (0.82) [−0.38, 0.21]	0.22 (0.62) [−0.01, 0.44]
WHOQOL	74.59 (5.38) [72.66, 76.53]	74.72 (4.60) [73.06, 76.38]

* *p* < 0.05.

** *p* < 0.01.

### 3.1 Sleep quality

**Figure 2:**
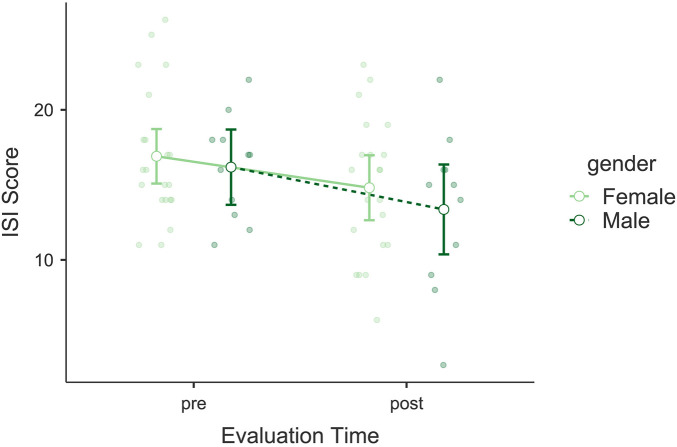
Estimated marginal means of the Insomnia Severity Index (ISI). Compares pre- and post-intervention ISI scores, showing significant reductions in insomnia severity.

The analysis revealed a significant decrease in Insomnia Severity Index (ISI) scores following the intervention. A significant main effect of *Evaluation Time* was observed, *F*(1, 30) = 12.465, *p* = 0.001, *η*p^2^ = 0.294, indicating an improvement in participants' sleep quality (see Figure 2). No significant interaction was found between *Evaluation Time* and *Gender*, *F*(1, 30) = 0.270, *p* = 0.607, *η*p^2^ = 0.009, nor was *Gender* a significant independent factor, *F*(1, 30) = 0.512, *p* = 0.480, *η*p^2^ = 0.017. *post hoc* comparisons showed a significant difference between pre- and post-intervention scores, *t*(30) = 3.53, *p*_bonf_ = 0.001. Bayesian model comparisons provided strong evidence in favor of the model including *Evaluation Time* over the null model (*BF*₁₀ = 26.61), supporting the presence of a pre-post change. The mean reduction in ISI scores was Δ*M* = −2.35 points. This change is below the minimal clinically important difference typically reported (≈5–6) and only shifts the group marginally from the “moderate” to the “subthreshold” insomnia range.

**Figure 3:**
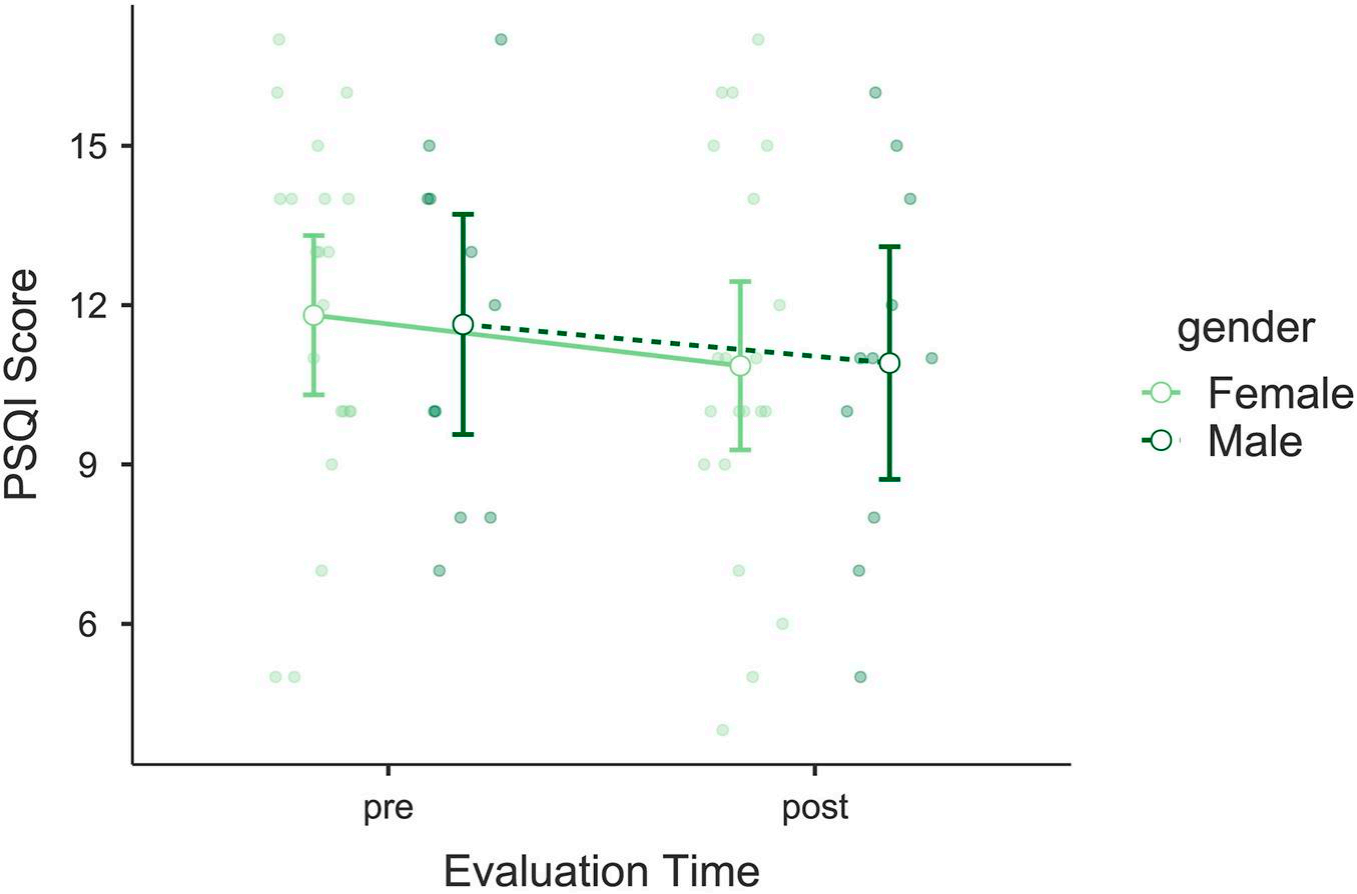
Estimated marginal means of the Pittsburgh Sleep Quality Index (PSQI). Compares pre- and post-intervention PSQI scores, showing significant improvements in overall sleep quality.

The analysis of the Pittsburgh Sleep Quality Index (PSQI) also showed significant improvement following the intervention. A significant main effect of *Evaluation Time* was found, *F*(1, 30) = 6.041, *p* = 0.020, *η*p^2^ = 0.168, indicating an improvement in the general sleep quality of the participants (see Figure 3). No significant interaction was found between *Evaluation Time* and *Gender*, *F*(1, 30) = 0.109, *p* = 0.744, *η*p^2^ = 0.004, nor was *Gender* a significant independent factor, *F*(1, 30) = 0.002, *p* = 0.961, *η*p^2^ < 0.001. *Post hoc* comparisons revealed a significant difference between pre- and post-intervention scores, *t*(30) = 2.46, *p*_bonf_ = 0.020. Bayesian model comparisons favored the model including *Evaluation Time* over the null model (*BF*₁₀ = 4.40), indicating moderate evidence for a pre–post change. The mean reduction in PSQI scores was Δ*M* = −0.87 points. This change does not reach the commonly used clinical threshold for meaningful improvement (≈3 points), and participants remain within the “poor sleeper” range (scores > 5).

### 3.2 Mental health related to sleep

**Figure 4:**
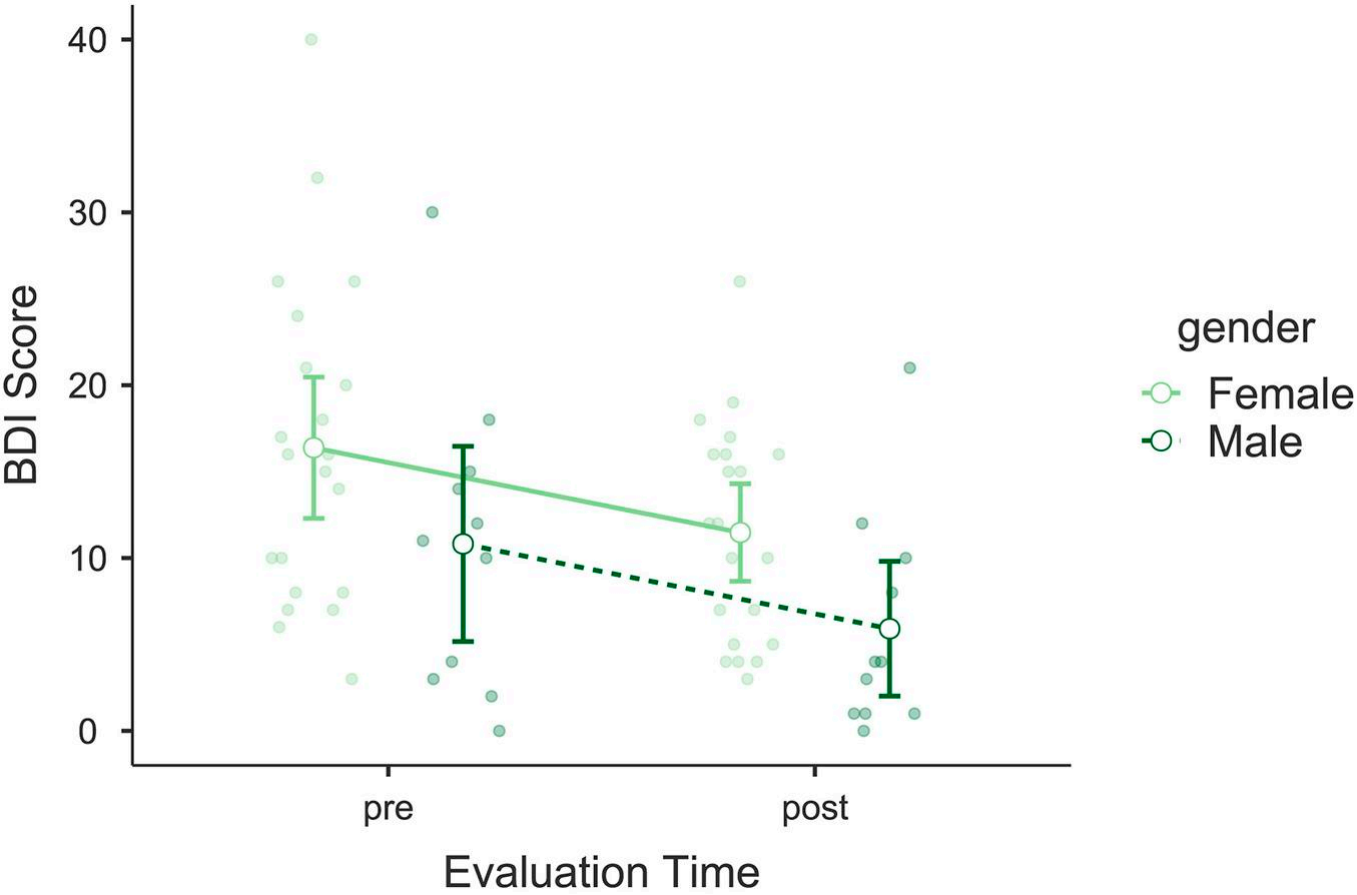
Estimated marginal means of the Beck Depression Inventory (BDI). Compares pre- and post-intervention BDI scores, showing significant reductions in depressive symptoms.

The analysis of the Beck Depression Inventory (BDI) showed a significant reduction in scores following the intervention. A significant main effect of *Evaluation Time* was observed, *F*(1, 30) = 13.0, *p* = 0.001, *η*p^2^ = 0.302, indicating a decrease in depressive symptoms among participants (see Figure 4). No significant interaction was found between *Evaluation Time* and *Gender*, *F*(1, 30) < 0.001, *p* = 0.999, *η*p^2^ < 0.001. However, *Gender* as an independent factor showed a significant effect, *F*(1, 30) = 4.58, *p* = 0.041, *η*p^2^ = 0.133. *post hoc* comparisons revealed a significant difference between pre- and post-intervention scores, *t*(30) = 3.60, *p*_bonf_ = 0.001, and between genders, *t*(30) = 2.14, *p*_bonf_ = 0.041, with men scoring lower than women. Bayesian model comparisons provided very strong evidence in favor of the model including *Evaluation Time* over the null model (*BF*₁₀ = 48.51), supporting the presence of a robust pre–post change. The mean reduction in BDI scores was Δ*M* = −4.91 points, corresponding to a shift across standard severity categories from mild to minimal depressive symptoms.

**Figure 5:**
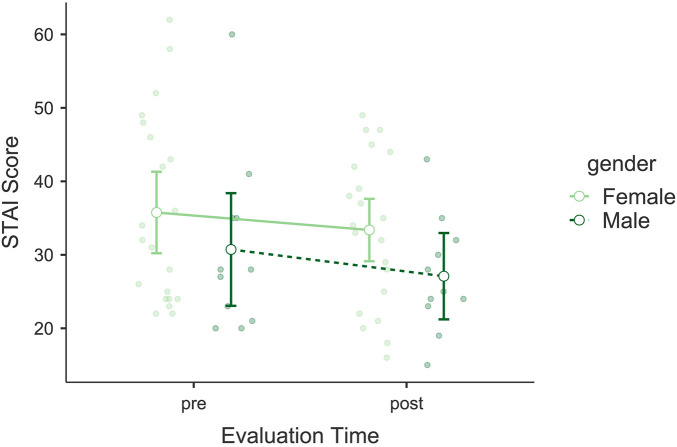
Estimated marginal means of the State-Trait Anxiety Inventory (STAI). Compares pre- and post-intervention anxiety scores, showing no significant changes in state anxiety levels.

The analysis of the State-Trait Anxiety Inventory (STAI) did not show significant differences in state anxiety scores following the intervention (see Figure 5). No significant main effect of *Evaluation Time* was observed, *F*(1, 30) = 2.048, *p* = 0.163, *η*p^2^ = 0.064, nor a significant interaction between *Evaluation Time* and *Gender*, *F*(1, 30) = 0.089, *p* = 0.767, *η*p^2^ = 0.003. *Gender* as an independent factor also did not show significant effects, *F*(1, 30) = 2.54, *p* = 0.121, *η*p^2^ = 0.078. Bayesian model comparisons favored the null model over the model including *Evaluation Time* (*BF*₁₀ = 0.58; *BF*₀₁ = 1.73), indicating anecdotal evidence for the absence of a pre–post change. The mean change in STAI-State scores from pre- to post-intervention was Δ*M* = −2.81 points. Both pre- and post-intervention means remained below the commonly used clinical cut-off for clinically significant anxiety (≥40).

**Figure 6:**
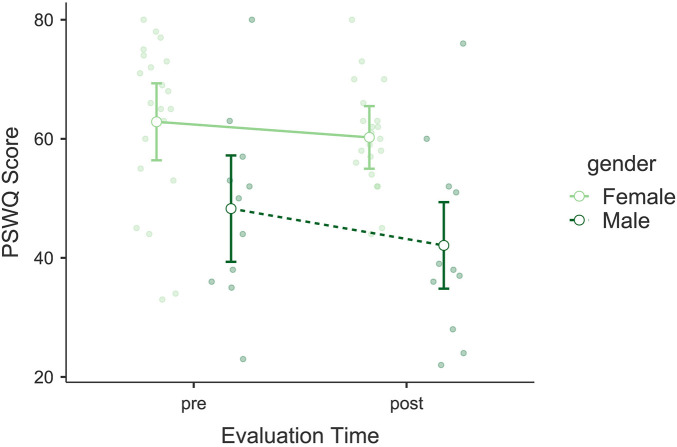
Estimated marginal means of the Penn State Worry Questionnaire (PSWQ). Compares pre- and post-intervention worry levels, showing significant reductions in participants' worry.

The analysis of the Penn State Worry Questionnaire (PSWQ) showed a significant reduction in scores following the intervention. A significant main effect of *Evaluation Time* was observed, *F*(1, 30) = 5.628, *p* = 0.024, *η*p^2^ = 0.158, indicating a reduction in participants' worry levels (see Figure 6). No significant interaction was found between *Evaluation Time* and *Gender*, *F*(1, 30) = 0.922, *p* = 0.345, *η*p^2^ = 0.003. However, *Gender* as an independent factor showed a significant effect, *F*(1, 30) = 12.9, *p* = 0.001, *η*p^2^ = 0.300. *Post hoc* comparisons revealed a significant difference between pre- and post-intervention scores, *t*(30) = 2.37, *p*_bonf_ = 0.024, and between genders, *t*(30) = 3.59, *p*_bonf_ = 0.001, with men scoring lower than women. Bayesian model comparisons showed only anecdotal evidence in favor of the model including *Evaluation Time* over the null model (*BF*₁₀ = 1.66), indicating weak support for a pre–post change. The mean reduction in PSWQ scores was Δ*M* = −3.84. Mean scores remained above the threshold for clinically significant worry (≥45) and below the threshold for severe pathological worry (≥60) at both time points.

### 3.3 Executive and cognitive function

**Figure 7:**
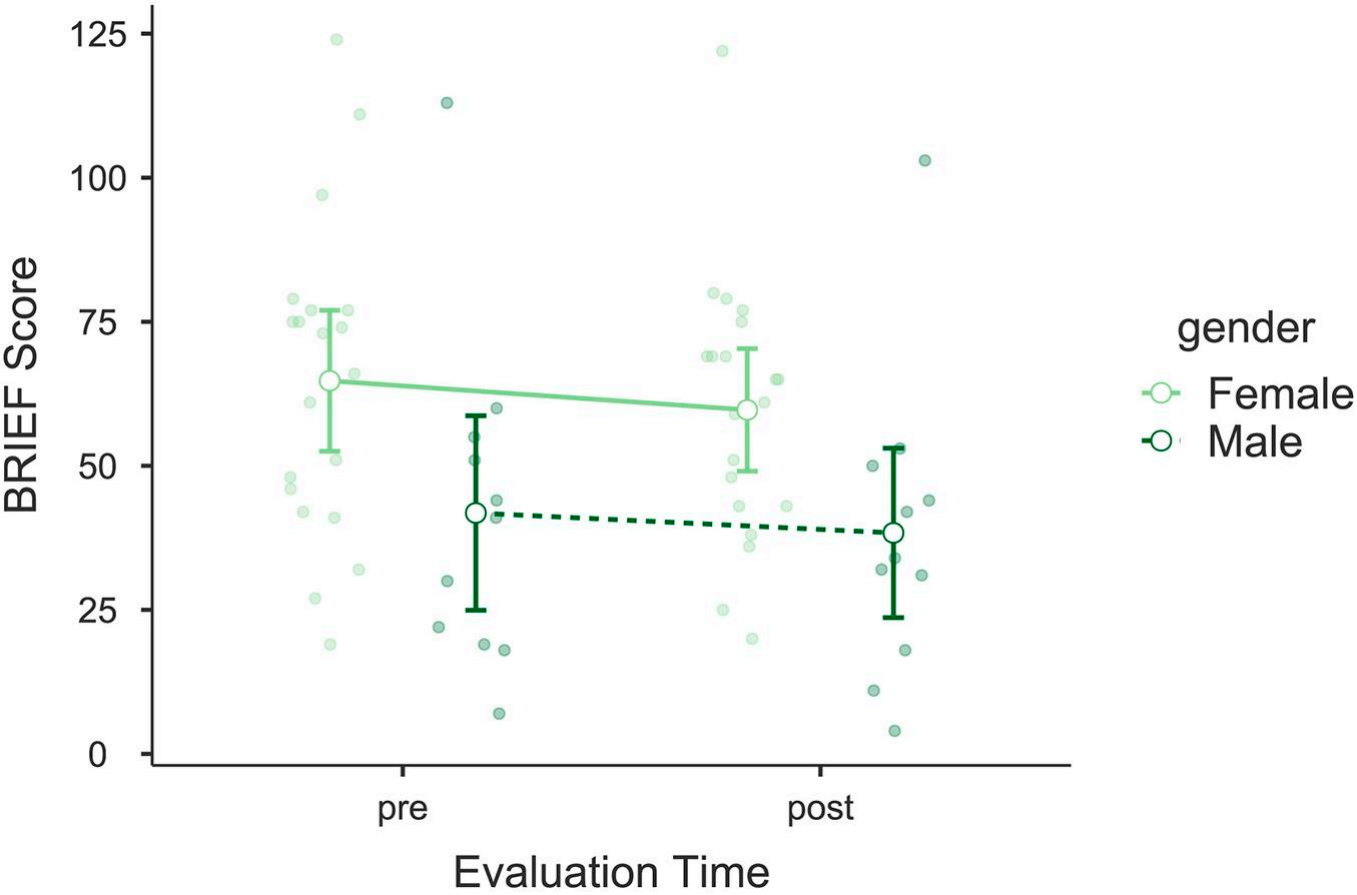
Estimated marginal means for the Behavior Rating Inventory of Executive Function (BRIEF). Compares pre- and post-intervention executive function scores, showing reduced cognitive interference with daily activities.

The analysis of the Behavior Rating Inventory of Executive Function (BRIEF) revealed a significant improvement in executive function scores following the intervention. A significant main effect of *Evaluation Time* was observed, *F*(1, 30) = 4.802, *p* = 0.036, *η*p^2^ = 0.138, indicating a reduction in BRIEF scores, which reflects decreased executive dysfunction and enhanced cognitive control (see Figure 7). While no significant interaction was found between *Evaluation Time* and *Gender*, *F*(1, 30) = 0.169, *p* = 0.684, *η*p^2^ = 0.006, *Gender* as an independent factor showed a significant effect, *F*(1, 30) = 5.58, *p* = 0.025, *η*p^2^ = 0.157. *post hoc* comparisons further confirmed a significant improvement from pre- to post-intervention *t*(30) = 2.19, *p*_bonf_ = 0.036, along with a significant gender difference, with men exhibiting lower scores than women, *t*(30) = 2.36, *p*_bonf_ = 0.025. Bayesian model comparisons provided anecdotal evidence in favor of the model including *Evaluation Time* over the null model (*BF*₁₀ = 2.66), suggesting a possible pre–post change, although the strength of evidence was limited. The mean change in total scores was Δ*M* = −4.50 points. Both pre- and post-intervention scores remained below the normative cut-off for clinically significant executive dysfunction (T ≥ 65).

**Figure 8:**
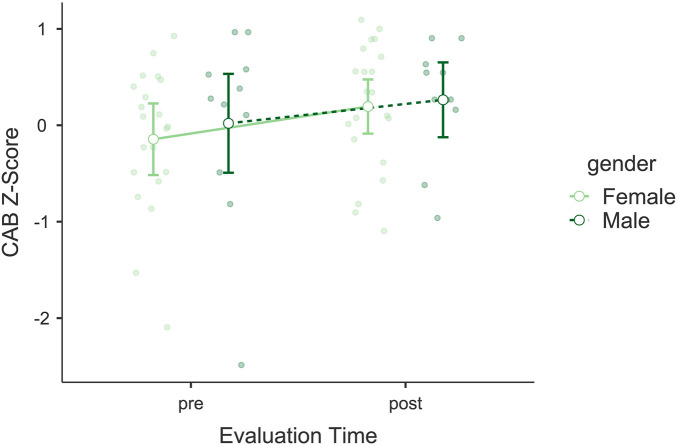
Estimated marginal means for the Cognitive Assessment Battery (CAB). Compares pre- and post-intervention cognitive performance scores, showing significant enhancements in memory and attention.

The analysis of the Cognitive Assessment Battery (CAB) indicated a significant improvement in cognitive performance following the intervention. A significant main effect of *Evaluation Time* was found, *F*(1, 30) = 13.228, *p* = 0.001, *η*p^2^ = 0.306, suggesting an enhancement in cognitive skills (see Figure 8). There was no significant interaction between *Evaluation Time* and *Gender*, *F*(1, 30) = 0.355, *p* = 0.556, *η*p^2^ = 0.012, and *Gender* as an independent factor did not show significant effects, *F*(1, 30) = 0.199, *p* = 0.659, *η*p^2^ = 0.007. *Post hoc* comparisons revealed a significant difference between pre- and post-intervention scores, *t*(30) = −3.64, *p*_bonf_ = 0.001. Bayesian model comparisons provided very strong evidence in favor of the model including *Evaluation Time* over the null model (*BF*₁₀ = 73.50), supporting a robust pre–post change. The mean increase in global CAB performance was Δ*M* = +0.31 *z*-score units. Both pre- and post-intervention values were within the normative range, above the threshold typically used to indicate clinically significant cognitive impairment (*z* ≤ −1.5).

### 3.4 Quality of life

**Figure 9:**
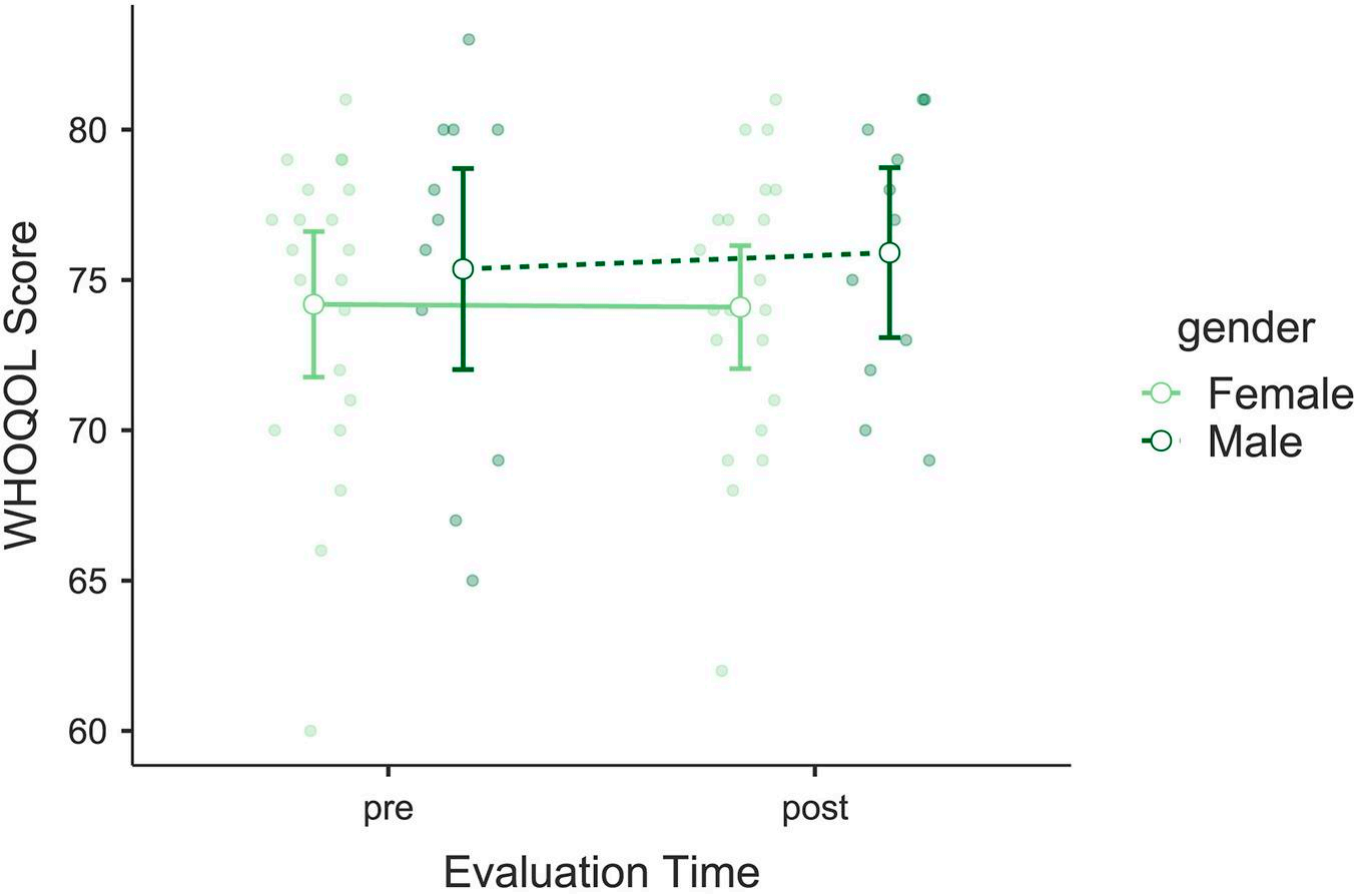
Estimated marginal means for the World Health Organization Quality-Of-Life Scale (WHOQOL). Compares pre- and post-intervention quality-of-life scores, showing no significant changes in overall life satisfaction.

The analysis of the World Health Organization Quality-of-Life Scale (WHOQOL) did not show significant changes in quality of life scores following the intervention. No significant main effect of *Evaluation Time* was found, *F*(1, 30) = 0.053, *p* = 0.820, *η*p^2^ = 0.002, indicating that the intervention did not significantly impact the overall quality of life of the participants (see Figure 9). Similarly, there was no significant interaction between *Evaluation Time* and *Gender*, *F*(1, 30) = 0.107, *p* = 0.746, *η*p^2^ = 0.004. *Gender* as an independent factor also did not show significant effects, *F*(1, 30) = 0.877, *p* = 0.356, *η*p^2^ = 0.028. Bayesian model comparisons provided moderate evidence in favor of the null model over the model including *Evaluation Time* (*BF*₀₁ = 3.88), indicating support for the absence of a pre–post change. The mean change in total scores was Δ*M* = +0.13 points. This change is below the minimal clinically important difference typically reported (≈2 points).

## 4 Discussion

This study examined whether engagement with a home-based computerized cognitive training (CCT) program is associated with changes in sleep-related, emotional, cognitive and quality-of-life outcomes in adults from the general population reporting persistent sleep difficulties. In line with the feasibility scope of the study, the primary aim was not to establish clinical efficacy, but to characterize the pattern, relative sensitivity, and coherence of multiple outcomes domains when a commercially available, self-guided cognitive training protocol is implemented in a naturalistic setting. From this perspective, the present findings provide information about which domains appear most responsive to short-term CCT exposure in a non-clinical population, and how these changes align with current conceptual models of insomnia that emphasize interactions between sleep regulation, daytime functioning, and affective state.

Across outcome domains, participation in the four-week program was associated with statistically reliable pre-post improvements in insomnia severity and subjective sleep quality, alongside clearer changes in measures of mood, worry, executive functioning, and cognitive performance. In contrast, state anxiety and overall quality of life remained largely stable over the intervention period. Importantly, the reduction in insomnia severity was sufficient to shift the group from the moderate to the subthreshold insomnia range, indicating a meaningful improvement in sleep status despite remaining below conventional minimal clinically important difference criteria. This transition is particularly relevant in a non-clinical community sample, where baseline symptom severity constrains the absolute magnitude of change that can be expected over a short intervention window. At the same time, when evaluated against established clinical benchmarks, changes in global sleep indices remained modest in absolute terms, while measures of state anxiety and quality of life showed no detectable change, likely reflecting floor effects associated with largely normative baseline levels. Together, this pattern suggests that short-term engagement with CCT may preferentially influence sleep-related complaints and general affective functioning, with more limited downstream impact on broader life-quality domains over a four-week period. Changes observed in emotional and cognitive measures further refine this interpretation. Reductions in BDI scores are best understood as reflecting improvements in overall mood state and emotional well-being rather than indicating an antidepressant effect, particularly given that mean baseline scores were in the mild range and shifted to the minimal/normal range post-intervention. Improvements in worry and executive functioning were smaller and more variable, and in some cases accompanied by stable between-group differences that accounted for more variance than the intervention itself. Cognitive performance showed reliable improvement despite baseline scores already falling within the normative range, suggesting enhanced efficiency rather than remediation of impairment. Within this framework, changes in cognitive and executive measures are most coherently interpreted as contributory or enabling processes that support daytime functioning and emotional regulation, which may in turn facilitate improvements in sleep-related outcomes, rather than as primary therapeutic targets in their own right.

When positioned within the broader insomnia intervention literature, this pattern is broadly consistent with prior studies reporting associations between CCT and improvements in subjective sleep and daytime functioning, while also underscoring important boundary conditions. Trials implementing longer training periods (typically eight weeks) and more structured or supervised delivery contexts have reported larger effects on sleep outcomes, in some cases extending to objective indicators such as actigraphy. For example, Haimov and Shatil (31) documented improvements across both subjective and objective sleep measures following extended cognitive training in clinically diagnosed older adults with insomnia, while community-based interventions with longer duration have similarly reported more robust sleep-related effects (32). Relative to those designs, the present study differed in three central respects that plausibly constrain the magnitude of sleep change: a shorter intervention dose, a fully home-based and self-guided format, and the inclusion of a non-clinically diagnosed sample with mild-to-moderate baseline disturbance. Notably, however, the convergence of effects on mood and cognitive functioning with earlier feasibility work using the same platform and task set (33) supports the conclusion that the training content itself can be reliably delivered via a commercial digital format and can engage relevant cognitive-affective processes even when sleep improvements remain subclinical.

Taken together, the present findings suggest that CCT engagement is associated with beneficial shifts in sleep complaints, mood state, and cognitive functioning in adults with persistent but non-clinical sleep difficulties, while also highlighting the limits of short-term intervention in producing clinically meaningful sleep remission. Improvements in insomnia severity were directionally consistent and clinically interpretable, yet modest in magnitude, reflecting both the baseline characteristics of the sample and the brief intervention window. Other domains, including anxiety and quality of life, remained stable, likely due to normative baseline functioning and the multidimensional, longer-term nature of these constructs. Thus, the current results should be interpreted as preliminary and hypothesis-generating, indicating that CCT may function as a supportive or protective intervention that helps maintain adaptive cognitive and emotional functioning and limits the escalation of sleep-related symptoms in otherwise healthy populations. Rather than positioning cognitive training in relation to established insomnia treatments, the present results support its feasibility as an independent, cognitively oriented approach worthy of further investigation in the context of sleep-related complaints (26, 27, 31).

While the primary focus of the present study was on overall pre-.post changes associated with CCT engagement, variability across individuals may further inform the interpretation of these effects. Accordingly, exploratory gender-related analyses were conducted given prior evidence of its relevance in sleep, cognitive, and emotional functioning (52, 53). Most outcomes did not reveal gender-based differences, yet significant effects emerged in three domains: worry, executive function, and depressive symptoms. Consistent with prior research, women exhibited higher levels of worry, a finding aligned with studies indicating that they tend to engage in greater rumination and elaborative processing of negative emotions compared to men (54). Similarly, higher BDI scores among women are in line with established literature suggesting that depressive symptoms are more frequently reported by women, potentially due to differences in affect regulation, stress responsiveness, or socialization factors (55). Likewise, men reported better executive functioning compared to women. While some studies suggest women's advantages in certain executive domains, findings on gender differences in executive function remain mixed and context-dependent (56, 57). Additionally, self-reported measures such as the BRIEF may be influenced by gender-related differences in self-perception rather than actual cognitive disparities (58). Given the unequal sample sizes and limited statistical power, these results should be interpreted with caution and viewed strictly as descriptive signals rather than evidence of robust gender effects.

From a neurobiological perspective, the present findings can be tentatively situated within two complementary theoretical frameworks that have been widely used to conceptualize insomnia, namely models emphasizing disruptions in functional connectivity—particularly within the default mode network (DMN)—and homeostatic models of sleep regulation. Insomnia has been associated with altered functional connectivity (FC) across large-scale networks involved in self-referential processing, attentional control, and emotional regulation, including the DMN, dorsal attention network, and frontoparietal control network (59, 60). Dysregulated DMN activity has been linked to persistent internal mentation and cognitive-emotional hyperarousal, which may interfere with the downregulation processes required for sleep initiation and maintenance (60, 61). Within this context, CCT may influence sleep-related outcomes indirectly by engaging executive control, working memory, and attentional processes during wakefulness, potentially reducing maladaptive cognitive activity associated with hyperarousal rather than directly targeting sleep mechanisms (62). In parallel, homeostatic models posit that cognitively demanding and novel tasks increase sleep pressure, reflected in subsequent changes in slow-wave sleep (SWS) and slow-wave activity (SWA), which play a central role in neural recovery and memory consolidation (27, 63–65). Experimental work has shown that sustained cognitive engagement can elevate SWA during subsequent sleep, consistent with a homeostatic response to increased cognitive load. From this perspective, short-term CCT exposure may contribute to sleep-related improvements by increasing daytime cognitive demand and promoting downstream homeostatic processes, while simultaneously modulating cognitive-affective activity linked to DMN hyperactivation (66–68). Importantly, the present study did not include objective sleep or neurophysiological measures, and these frameworks are therefore invoked to contextualize the observed behavioral and self-report changes rather than to propose specific mechanisms. Direct evidence linking CCT to changes in functional connectivity, DMN dynamics, or SWS/SWA remains limited, and future studies combining cognitive training with EEG or neuroimaging will be required to clarify whether these processes differentially contribute to treatment responsiveness across insomnia phenotypes (69), ideally incorporating longer intervention periods given that a four-week window may be insufficient to detect structural or functional neurobiological adaptation.

The study has several limitations that should be considered when interpreting the results. Firstly, no longitudinal follow-up was conducted, preventing the evaluation of long-term effects of the cognitive intervention on sleep quality and general well-being. Additionally, the observed improvements may have been partially influenced by personalized follow-up and periodic contact with the research team, which could have introduced expectancy or engagement-related bias. Another limitation is the exclusive reliance on subjective measures to assess sleep quality and psychological functioning. Although these instruments are validated and widely used, the absence of complementary objective assessments such as actigraphy, polysomnography, or neuroimaging limits our ability to determine whether the reported changes correspond to physiological or neural alterations associated with sleep and cognition. Such objective measures could reveal patterns not detectable through self-report alone; for instance, actigraphy or polysomnography may show improvements—or the absence thereof—in sleep architecture even when subjective sleep quality remains unchanged (70), while neuroimaging approaches could clarify whether cognitive or emotional changes are accompanied by modifications in functional connectivity (71). Furthermore, the time of day at which participants completed the cognitive training was not controlled. Although prior studies have reported comparable effects when training is conducted in the morning (23) or shortly before sleep (27), this factor may nonetheless have contribute to unexplained variability in the present sample. In addition, the multidomain nature of the training protocol precludes isolating the contribution of specific cognitive functions to the observed outcomes. A central methodological limitation concerns the lack of a control group combined with a modest sample size. Without a comparison condition, it is not possible to determine to what extent the observed changes reflect intervention-related effects rather than expectancy effects, spontaneous remission, regression to the mean, or other uncontrolled variables. Accordingly, the present single-group pre–post design does not allow for causal inferences regarding the efficacy of the CCT program, and the findings should therefore be interpreted as preliminary and hypothesis-generating. Finally, the relatively high dropout rate observed during the intervention constitutes an additional limitation. The requirement of approximately 45 min of daily training represents a substantial time commitment and may have contributed to attrition. Because this feasibility study did not include an intention-to-treat analysis, the results reflect only participants who completed the full protocol and may therefore overrepresent individuals with higher motivation or availability, limiting ecological validity and the estimation of real-world adherence. Future studies should consider lower-intensity or more flexible training schedules and incorporate randomized controlled designs with intention-to-treat analyses to better assess feasibility, adherence, and efficacy at scale.

Nevertheless, these limitations do not diminish the promise of CCT as a complementary, non-pharmacological intervention for insomnia prevention and management. Its accessibility, adaptability, and cost-effectiveness make it an attractive option, particularly for individuals who may have difficulty accessing in-person therapies or who prefer self-guided, flexible interventions. The gamification of cognitive tasks not only makes the training more engaging and motivating for users but also can improve treatment adherence, a common challenge in long-term interventions. Furthermore, the ability to personalize the training based on individual needs and performance ensures that the intervention is adaptive and effective. Lastly, as a non-pharmacological intervention, it avoids potential side effects associated with medication and can be easily integrated into multimodal treatment programs.

## 5 Conclusions

The present findings suggest that participation in a computerized cognitive training (CCT) program was associated with modest improvements in subjective sleep quality, cognitive performance, and selected emotional outcomes in adults reporting habitual sleep difficulties. However, given the single-group pre–post design, the small sample size, and the absence of a control condition, these associations cannot be interpreted as evidence of intervention efficacy or causal effects. The lack of parallel changes in quality of life over the four-week period further indicates that the observed effects were limited in scope and magnitude. Rather than demonstrating clinical effectiveness, the results should be viewed as preliminary and hypothesis-generating, supporting the feasibility of delivering an intensive, home-based CCT protocol in a non-clinical population. Future research should rely on adequately powered randomized controlled trials, include objective sleep measures and longer follow-up periods, and directly compare CCT with established interventions such as CBT-i to determine whether and under which conditions cognitive training may provide added value for individuals with persistent sleep complaints.

## Data Availability

The datasets presented in this study can be found in online repositories. The names of the repository/repositories and accession number(s) can be found below: https://doi.org/10.17605/OSF.IO/WEHDS.
