# Supplementary material for: Cognitive training at home for clinically defined insomnia: effects on sleep and psychological functioning
Source: Front Digit Health. 2026 Feb 2;8:1725773. doi: 10.3389/fdgth.2026.1725773 (PMC12907364; doi:10.3389/fdgth.2026.1725773)
Supplement: Supplementary file 1 [file Table1.docx]

Supplementary Material

# S1. Brain training games used

| **Name** | **Overview** | **Cognitive skills trained** |
| --- | --- | --- |
| Candy Factory | Players introduce sweets into corresponding paper bags and identify which of the sweets shown on each bag matches the ones saved. | Recognition  Visual Short-Term Memory  Short-Term Memory  Phonological Short-Term Memory |
| Candy Line Up | Players fill vases with the same type of candy, only placing identical candies on top of each other | Mental Planning  Working Memory  Updating |
| Color Frenezy | Players select tiles in different shades, from lightest to darkest, and identify groups with the largest or smallest area. | Visual Perception  Processing Speed  Focused Attention |
| Color Rush | Players drive a vehicle through a race track, collecting energy balls of the same color while avoiding obstacles. | Updating  Reaction Time  Shifting (Cognitive Flexibility)  Estimation |
| Crossroads | Players keep balls from colliding by placing rocks at intersections to prevent crashes. | Divided Attention  Estimation  Updating |
| Drive me crazy | Players follow written signals and click on the blue arrows in the same order as indicated, ignoring distractions. | Cognitive Flexibility  Auditory Perception  Contextual Memory  Inhibition |
| Fresh Squeeze | Players locate and connect pipes to guide liquid to a monster's mouth. | Spatial Perception  Planning  Updating |
| Fuel a Car | Players attend to the needs of each vehicle, such as pouring gasoline, and then raise the barrier to avoid queues. | Divided Attention  Response Time  Processing Speed |
| Mandala | Players create intricate and colorful designs. | Visual Perception  Planning  Visual Short-Term Memory |
| Math Madness | Players memorize an image with an associated value and solve a mathematical operation, then remember a series of images and their positions to solve another operation. | Auditory Short-term Memory  Recognition  Non-verbal Memory  Short-term Memory |
| Math Twins | Players join pairs of numbers to achieve a target sum. | Visual Scanning  Spatial Perception  Focused Attention |
| Melody Mayhem | Players find pairs containing the same melodies. | Phonological Short-term Memory  Auditory Perception  Recognition |
| Minus Malus | Players use a cannon to launch balls with assigned values to knock down falling packages, matching the target points on the packages. | Processing Speed  Shifting (Cognitive Flexibility)  Working Memory |
| Neuron Madness | Players collect neurons in a specified order while avoiding collisions and color changes. | Focused Attention  Visual Scanning  Inhibition |
| Numbers Line | Players are presented with a target number and must shoot balls onto a number line to reach that target. | Visual Scanning  Processing Speed  Short-term Memory |
| Perfect Tension | Players remove pieces in the correct order to prevent others from falling into the vacuum. | Updating  Planning  Spatial Perception |
| Piece Making | Players memorize three totem figures and then find the same set on one of the stones below. | Visual Short-Term Memory  Visual Perception  Recognition |
| Reaction Field | Players hit the target mole while avoiding others, including those with dynamite attached. | Inhibition  Response Time  Shifting (Cognitive Flexibility) |
| Space Rescue | Players rescue astronauts while dodging obstacles and remembering objects in the background. | Contextual Memory  Response Time  Spatial Perception  Estimation |
| Sudoku | Players fill in gaps with numbers, ensuring no repetition in rows, columns, or subgrids. | Planning  Spatial Perception  Inhibition  Phonological Short-Term Memory |
| Traffic Manager | Players control traffic lights to allow some vehicles through intersections while preventing others from causing accidents. | Divided Attention  Estimation  Planning  Updating |
| Visual Crossword | Players remember the name of an object shown in an image and select the first letter of the word from given options. | Working Memory  Visual Perception  Naming |
| Water Lilies | Players memorize and reproduce the order in which stimuli are illuminated. | Visual Short-Term Memory  Non-verbal Memory  Planning  Short-term Memory  Working Memory |
| Words Birds | Players move stimuli to form the name of the object presented using the letters in each row and column. | Updating  Naming  Visual Scanning |
